# Basal Serum Neurokinin B Levels in Differentiating Idiopathic Central Precocious Puberty from Premature Thelarche

**DOI:** 10.4274/jcrpe.3817

**Published:** 2017-06-01

**Authors:** Mesut Parlak, Doğa Türkkahraman, Hamit Yaşar Ellidağ, Gamze Çelmeli, Ayşe Eda Parlak, Necat Yılmaz

**Affiliations:** 1 Antalya Training and Research Hospital, Clinic of Pediatric Endocrinology, Antalya, Turkey; 2 Antalya Training and Research Hospital, Clinic of Biochemistry, Antalya, Turkey; 3 Akdeniz University Faculty of Medicine Hospital, Clinic of Pediatric Endocrinology, Antalya, Turkey; 4 Antalya Training and Research Hospital, Clinic of Radiology, Antalya, Turkey

**Keywords:** Neurokinin B, Kisspeptin, Precocious puberty

## Abstract

**Objective:**

To find out the diagnostic role of kisspeptin and neurokinin B in idiopathic central precocious puberty (ICPP) and premature thelarche (PT).

**Methods:**

The girls who presented with early breast development before the age of 8 years were evaluated. Patients with intracranial pathologies were excluded. Basal and stimulated follicle-stimulating hormone/luteinizing hormone (LH) levels and basal neurokinin B/kisspeptin levels were measured. Patients who had peak value of LH >5 mIU/mL and a bone age (BA)/chronological age (CA) ratio >1.1 were diagnosed as central precocious puberty (CPP), while cases who did not meet these criteria were diagnosed as PT. Healthy age-matched prepubertal girls were included as the control group.

**Results:**

The study group contained 25 girls with ICPP (7±0.8 years), 35 girls with PT (6.8±0.7 years), and 30 controls (6.7±0.7 years). Basal serum kisspeptin and neurokinin B levels were 2.36±0.47 ng/mL and 2.61±0.32 ng/mL, respectively in the ICPP group, 2.23±0.43 ng/mL and 2.24±0.23 ng/mL, respectively in the PT group, and 1.92±0.33 ng/mL and 2.03±0.24 ng/mL, respectively in the controls. Both kisspeptin and neurokinin B levels were higher in the ICPP and PT groups compared to controls (p<0.05). Moreover, basal neurokinin B level was different between ICPP and PT groups (p<0.01). A serum neurokinin B level of 2.42 ng/mL provided the most appropriate level to differentiate ICPP from PT, with a sensitivity of 84% and specificity of 77.1%.

**Conclusion:**

Differentiation of CPP from PT is sometime difficult, and there is a need for a simple method for the differential diagnosis. Our results suggest that basal serum neurokinin B level can be used as an adjunctive parameter to differentiate ICCP from PT.

## What is already known on this topic?

Kisspeptin and neurokinin B have an important role in regulation of puberty. Differentiation of central precocious puberty (CPP) from premature thelarche (PT) is sometime difficult.

## What this study adds?

Kisspeptin and neurokinin B levels were higher in the idiopathic CPP and PT groups compared to controls. We demonstrated that serum neurokinin B level can be used as an adjunctive parameter to differentiate idiopathic CCP from PT.

## INTRODUCTION

Puberty is a phase of the maturation process during which secondary sex characteristics develop and reproductive capacity is attained. The activation of pulsatile gonadotropin-releasing hormone (GnRH) secretion from hypothalamic neurons is a major event in the onset of puberty. However, mechanism and timing of GnRH secretion at puberty have not been explained clearly as yet.

Kisspeptin, neurokinin B, and dynorphin A (KNDy) are co-expressed in neurons of the arcuate nucleus of the hypothalamus and they play an important role in regulating pulsatile GnRH secretion (1). Kisspeptin is a neuropeptide encoded by the KISS1 gene. KISS and G-protein coupled receptor-54 (GPR54) signaling complex have been recognized as essential regulators of pubertal activation (2). In humans, neurokinin B and its receptor are encoded by the *TAC3* and *TAC3R* genes, respectively. Recent studies have shown that neurokinin B can affect GnRH neuronal activity directly and indirectly. Kisspeptin is the most potent secretagogue for GnRH, while neurokinin B stimulates kisspeptin to initiate GnRH pulse (3,4).

Premature activation of GnRH secretion causes early puberty including central precocious puberty (CPP) and premature thelarche (PT). CPP is defined as development of secondary sex characteristics in girls before 8 years of age with an acceleration of linear growth, rapid bone maturation, and axillary and/or pubic hair development. On the other hand, in girls, PT is defined as isolated breast development before age of 8 years with no other signs of puberty (5). Differentiation of CPP from PT is often difficult in the early stage. Therefore, there is a need for a simple method for the differential diagnosis. To date, numerous studies have been published on kisspeptin and/or neurokinin B levels in girls with CPP (6,7,8). In these studies, it has been demonstrated that basal serum kisspeptin and/or neurokinin B levels are higher in girls with CPP and PT as compared to healthy controls. However, none of these studies have found a clear correlation in the differential diagnosis of CPP and PT.

In this study, we compare the basal serum neurokinin B and kisspeptin levels in the differential diagnosis of idiopathic CPP (ICPP) and PT.

## METHODS

### Patients and Methods

Girls who had been referred to our clinic between May 2013 and December 2014 with a complaint of early breast development before the age of 8 years were evaluated. Height and weight were measured using a stadiometer and a calibrated electronic scale, respectively. Height standard deviation scores (H-SDS) and body mass index (BMI) SDS were calculated according to Turkish reference values (9). The same qualified pediatric endocrinologist assessed the pubertal stages in all subjects. An x-ray of the left wrist was taken, and assessment of the bone age (BA) was done by the same person according to the method of Greulich and Pyle.

CPP is defined as development of secondary sex characteristics before age 8 years in conjunction with a BA/chronological age (CA) ratio >1.1 and a peak luteinizing hormone (LH) value >5 mIU/mL after GnRH stimulation. Cranial magnetic resonance imaging (MRI) was performed in all subjects diagnosed as CPP. Those without any organic cranial pathology were classified as ICPP and included in the study. Subjects whose BA/CA ratio was ≤1.1 and/or peak LH level ≤5 mIU/mL were considered as PT (5).

Exclusion criteria were having history of exogenous exposure to estrogen, obesity (BMI-SDS ≥2), organic brain disease, mental retardation, primary gonadal or adrenal diseases, or any other endocrine disease. The control group consisted of age-matched healthy prepubertal girls without obesity.

The written informed consent was obtained from the parents, and the study was approved by the local ethics committee.

### Hormonal Evaluation

GnRH stimulation test was performed in all patients with early breast development. GnRH (Gonadorelin acetate, Ferring) was administered intravenously (2.5 mcg/kg, max 100 mcg), and samples were taken at 30, 60, and 90 minutes after injection.

Basal and stimulated levels of serum LH, follicle-stimulating hormone (FSH) and estradiol levels were measured using Beckman Coulter, a two-site immunoenzymatic (sandwich) assay, and an auto analyzer.

### Measurement of Neurokinin B and Kisspeptin

The blood samples were drawn in the morning (8-9 AM) from patients and controls. Neurokinin B and kisspeptin levels were measured using a commercially available ELISA kit (Phoenix Pharmaceutical, California, USA, and USCN Life Science, Texas, USA, respectively). Neurokinin B assay range was 0-100 ng/mL, and kisspeptin assay range was 0.06-4 ng/mL (after 1:1 dilution with sample diluent for kisspeptin). For both assays, intrassay coefficient of variation (CV) was <10%, and the interassay CV was <12%. The assays employed the quantitative sandwich enzyme immunoassay technique. In order to avoid variation within an assay, measurements were performed twice using the same ELISA kit.

### Statistical Analysis

The SPSS 20.0 software program was used for statistical analysis. Shapiro-Wilk test was used for testing normality. Groups were compared using ANOVA and Tukey’s HSD (Honestly Significant Difference) test or Kruskal-Wallis test, later the groups were compared with one another using Mann-Whitney U-test and Bonferroni correction. A p-value of less than 0.05 was considered as statistically significant. A receiver operating characteristic-area under curve (ROC-AUC) was constructed to obtain neurokinin B levels to differentiate between ICPP and PT groups. The sensitivity and specificity were calculated based on cut-off points obtained by the ROC curve.

## RESULTS

The study group contained 25 girls with ICPP (mean age 7.02±0.79 years), 35 girls with PT (mean age 6.85±0.7 years), and 30 healthy prepubertal controls (mean age 6.74±0.73 years). Age, H-SDS, and BMI-SDS values were similar in all three groups (p>0.05). Basal serum LH, and peak serum LH and peak LH/FSH ratio, BA and BA/CA ratio were significantly higher in the ICPP group compared to the PT group (p<0.05).

**Figure 1:**
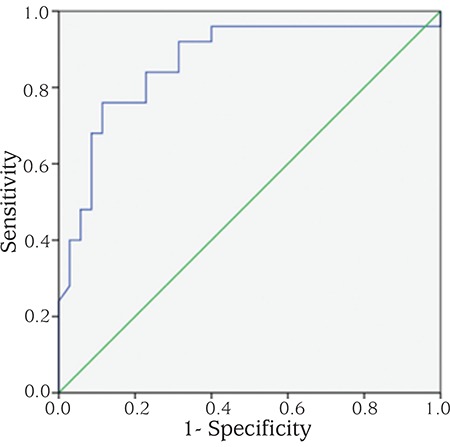
The receiver operating characteristic curve of neurokinin B level. The area under curve was 0.86 [95% confidence interval (CI): 0.75-0.93]. The optimal serum neurokinin B cut-off value to differentiate idiopathic central precocious puberty from premature thelarche was 2.42 ng/mL with a sensitivity of 84% (95% CI: 63.9-95.5) and specificity of 77.1% (95% CI: 59.9-89.6)

Serum kisspeptin and neurokinin B levels were higher in girls with ICPP and PT compared to controls. While serum neurokinin B level was higher in the ICPP group compared to PT group (p=0.001), no significant difference was found in serum kisspeptin level between ICPP and PT groups (p>0.05) (Table 1). An ROC-AUC was constructed to obtain neurokinin B levels to differentiate between ICPP and PT groups; a serum neurokinin B level of 2.42 ng/mL provided the most appropriate level with a sensitivity of 84% [95% confidence interval (CI): 63.9-95.5] and a specificity of 77.1% (95% CI: 59.9-89.6) (Figure 1).

## DISCUSSION

The control of onset of puberty is regulated by a network which includes KNDy neurons. These sex-steroid responsive neurons communicate via project ipsi- and contralaterally to themselves with neuropeptides (4). KNDy neuropeptides have been described as gatekeepers of puberty in regulating pulsatile GnRH secretion (1,2).

Kisspeptin is the most extensively studied neuropeptide in puberty and precocious puberty (6,7,8). In human, *KISS1* is located on chromosome 1q32 and is detected in brain, pancreas, placenta, testis and genital tract (10). Physiological studies demonstrated that kisspeptin and its receptor GPR54 are essential for GnRH neuron function. Kisspeptin/GPR54 system can directly stimulate GnRH cells in the arcuate nucleus of hypothalamus (11). Kisspeptin is a major stimulant for GnRH secretion with GPR54 coupling and has an important role in induction of puberty (12). In some previous studies, serum kisspeptin level has been studied in ICPP and PT cases. De Vries et al (13) investigated the serum kisspeptin level in 31 girls with ICPP and reported significantly higher level of kisspeptin in girls with ICPP than that of age-matched prepubertal girls. In the same study, there was no correlation between kisspeptin and peak LH level in the ICPP group. In another study of 30 girls with ICPP by Rhie et al (7), it was shown that the serum kisspeptin level in ICPP patients was higher than that in prepubertal controls. In both studies, authors suggested that basal kisspeptin level is a useful diagnostic tool in diagnosis of ICPP. Yang et al (6) reported that kisspeptin levels in ICPP patients were higher than those in patient with PT and in prepubertal controls. After 6 months of treatment with GnRHa, kisspeptin level was lower than that prior to treatment. In another study, Abacı et al (8) evaluated serum kisspeptin levels in girls with ICPP and PT compared to healthy prepubertal girls. They detected higher serum kisspeptin levels in ICPP and PT groups than in controls, but there was no significant difference in serum kisspeptin levels between ICPP and PT groups. Similarly, in the present study, kisspeptin level was higher in the girls with ICPP and PT than in controls, while there was no significant difference between ICPP and PT groups. Our study confirms the findings of previous studies, showing that serum kisspeptin level increases in early puberty and is a useful parameter in diagnosis of ICPP but is not helpful to differentiate ICCP from PT.

In recent years, kisspeptin has been accepted as an important indicator of pubertal onset. However, De Croft et al (14) showed that almost all arcuate kisspeptin neurons were directly activated by substance P and neurokinin A and B. True et al (15) suggest that amplification of neurokinin B secretion drives the release of kisspeptin from KNDy neurons. Neurokinin B (TAC3) and TAC3R are known to be expressed mainly in hypothalamic neurons and widely expressed in peripheral tissues (3,16). Additionally, TAC3R mRNA has been identified in GnRH axons (17), and it has been shown that neurokinin B peptides are in direct contact with GnRH axons and directly induce GnRH secretion from TAC3R-expressing GnRH neurons (18,19).

To our knowledge, there is only one reported study evaluating the serum neurokinin B levels in girls with ICPP and PT compared to prepubertal controls (8). In this study, Abacı et al (8) reported that neurokinin B is significantly higher in ICPP and PT groups than in age-matched prepubertal controls, while there is no difference between the ICPP and PT groups. The limited number of subjects (22 girls with ICPP and 20 with PT) was reported as the limitation of the study.

As a very similar study, we evaluated serum neurokinin B levels in 25 girls with ICPP and in 35 girls with PT compared to 30 healthy prepubertal girls. In contrast to Abacı et al’s study (8), we excluded obese patient from the study as obesity by itself can trigger puberty via leptin or other unknown neuropeptides. Additionally, we used a BA/CA ratio >1.1 as a parameter in the differential diagnosis of ICPP. We think that these important criteria make our results more acceptable compared to Abacı et al’s study (8). In our study, we detected higher serum neurokinin B levels in girls with ICPP and PT than in controls. Moreover, serum neurokinin B level was higher in the ICPP group compared to the PT group. A serum neurokinin B level of 2.42 ng/mL was found as the most appropriate level in differentiating ICPP from PT, with a sensitivity of 84% and specificity of 77.1%. Although the percentages of sensitivity and specificity are not too high, we think that serum neurokinin B level can be used as an adjunctive parameter, in addition to GnRH stimulation test, to differentiate ICCP from PT. Nevertheless, the assay should be validated in larger cohorts.

In conclusion, in this study, we examined the role of kisspeptin and neurokinin B in ICPP and PT patients. Given the fact that differentiation of CPP from PT is sometime difficult and the need for a simple method for the differential diagnosis, we suggest that basal serum neurokinin B level can be used as an adjunctive parameter to differentiate ICCP from PT.

## Figures and Tables

**Table 1:**
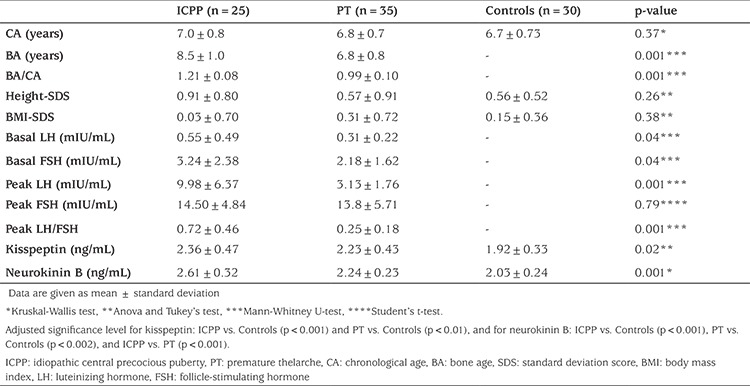
Clinical and laboratory characteristics of the study group
